# A computed tomography–based morphometric analysis of thoracic pedicles in a European population

**DOI:** 10.1186/s13018-024-05171-3

**Published:** 2024-10-17

**Authors:** Alberto Alfieri Zellner, Christian Prangenberg, Jonas Roos, Soufian Ben Amar, Tamara Babasiz, Christopher Wahlers, Peer Eysel, Johannes Oppermann

**Affiliations:** 1 Klinik und Poliklinik für Orthopädie und Unfallchirurgie, Universitätsklinikum Bonn, Venusberg Campus 1, 53127 Bonn, Deutschland; 2 Klinik und Poliklinik für Orthopädie, Unfallchirurgie und Plastische-Ästhetische Chirurgie, Uniklinik Köln, Joseph-Stelzmann-Str. 24, 50931 Köln, Deutschland

**Keywords:** Spine surgery, Spine anatomy, Computed tomography imaging, Pedicle morphology

## Abstract

**Purpose:**

The goal of this retrospective study was to perform a CT imaging assessment of thoracic pedicles to provide a representative understanding of pedicle morphology for pedicle-based fixation systems commonly used in orthopedics, trauma and neurosurgery. This study aimed to better understand the morphology of the spine and give spine surgeons a better understanding of thoracic spine anatomy.

**Methods:**

In this study, we retrospectively measured the thoracic spine pedicles of a total of 16 males and 16 females, totaling in 768 individual pedicles. For the measurements, we used standardized planes in computed tomography imaging with a maximum slice thickness of 1 mm.

**Results:**

In brief, we identified significant differences in various measurements of male and female pedicle morphology. The medial cortical wall of the pedicles was significantly thicker than the lateral wall, and, in both sexes, the thoracic vertebral body number four was the vertebra with the least amount of cortical bone in the pedicle.

**Conclusions:**

Surgeons performing operations involving pedicle screw placement should be aware of the sex-specific differences in thoracic spine pedicle morphology noted in this research.

## Introduction

The intricate nature of the thoracic spine presents unique challenges for orthopedic surgeons specializing in spinal interventions, particularly those involving pedicle screw fusion. As surgical techniques evolve and demand precision, a profound understanding of thoracic spine morphology becomes paramount for optimizing patient outcomes, minimizing complications, and advancing the field of spinal orthopedics. The anatomy, neighboring structures and gender specific morphology have previously been described in a plethora of studies [1–5]. It should be noted that it is well known that females have significantly smaller sized pedicle diameters compared to males, and that, on average, the medial pedicle wall is thicker and should therefore be used for orientation while inserting pedicle screws. To our knowledge, no studies have been published regarding European cohorts so far.

Surgical intervention may be indicated after trauma or subsequent degenerative or deformity changes in the thoracic spine. The indications for surgical treatment include failed conservative treatment, vertebral instability, impaired sensory or motor functions, and tumors. A vast number of scores and classifications exist that can be collected preoperatively (for example “Bauer Score”, “Modified score for therapeutic decision making in OF”, “AO Spine Thoracolumbar Injury Classification System and Treatment Algorithm”) to ease the decision-making process [6–9]. A common type of surgery performed in the thoracic spine is the spinal fusion (or temporary dorsal stabilization respectively), which works through screw placement in the pedicles and has been established for a long time [10–12]. The indications for this type of surgery are mostly deformities, fractures, and degenerative diseases of the spine. This kind of surgery aims to stabilize the spine by fusing two or more vertebrae together. In degenerative cases, this fusion aims to restore the physiological axis, rotation, and inclination of the spine and decrease motion and therefore pain in the affected segment. In fractures, the pedicle screws build the base for the fixateur interne that bridges the affected segment that allows it to heal. In either case, a fundamental understanding of pedicle morphology is crucial for obtaining optimal results. For these kinds of surgeries, long-term benefits are difficult to achieve. Screw placement is crucial for obtaining benefits and avoiding complications. Different screw placement techniques, such as the costotransverse screw technique, have been analyzed but seem to be inferior compared to the standard pedicle screw fixation [13]. Since the introduction of computed tomography (CT)-navigation systems for intraoperative use, screw placement has been shown to be more accurate [14–16]. Pitfalls during the use of CT-navigation include morphological changes in the anatomy and reference CT images, imprecise calibration of the system while using the wrong registration points and streak artifacts in the imaging (especially in patients with inlying metal implants). Complications include injury to neurovascular structures. CT navigation has helped to reduce complications created by pedicle perforation during screw fixation [17].

The rationale behind this study is rooted in the imperative to bridge the existing knowledge gap for a European cohort. We aimed to enhance the surgical decision-making process for this cohort. By meticulously characterizing the intricacies of thoracic spine morphology, we aspire to offer orthopedic surgeons a nuanced perspective that goes beyond traditional anatomical textbooks. Our findings aimed to elucidate the potential challenges and pitfalls encountered during pedicle screw fusion procedures, providing surgeons with the knowledge to navigate the complexities of the thoracic spine with heightened precision. With this study we hope to improve the current understanding of the morphology of the thoracic spine pedicles and hope to give spine surgeons a better understanding of thoracic pedicle anatomy. Furthermore, correct long-term screw placement is very important, since many of these operations are only reversible with considerable operative effort. Younger individuals who undergo these surgeries automatically imply individuals live with screws for a longer amount of time.

## Materials and methods

**Fig. 1:**
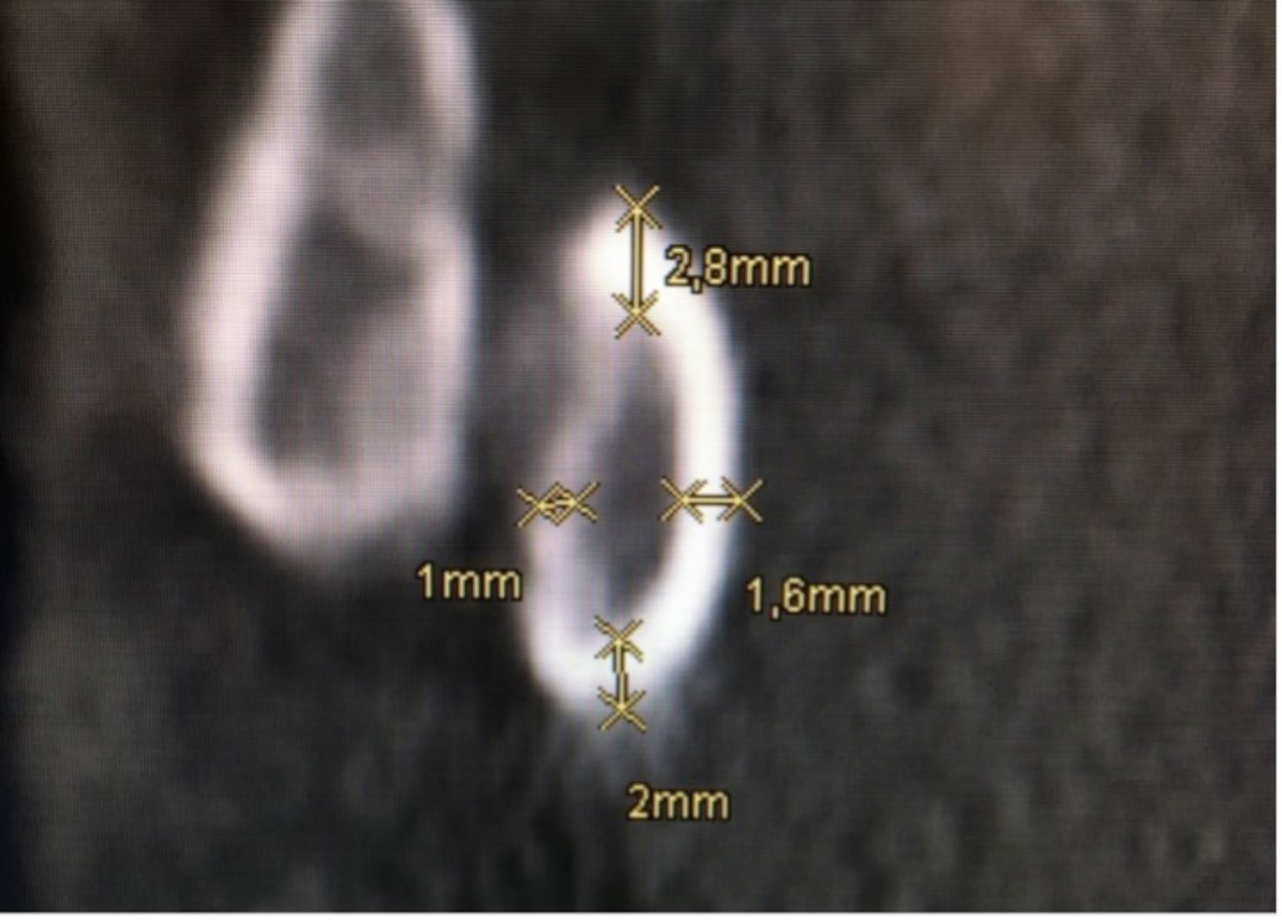
Measurement of cortical thickness of the thoracic pedicle in the coronar plain

**Fig. 2:**
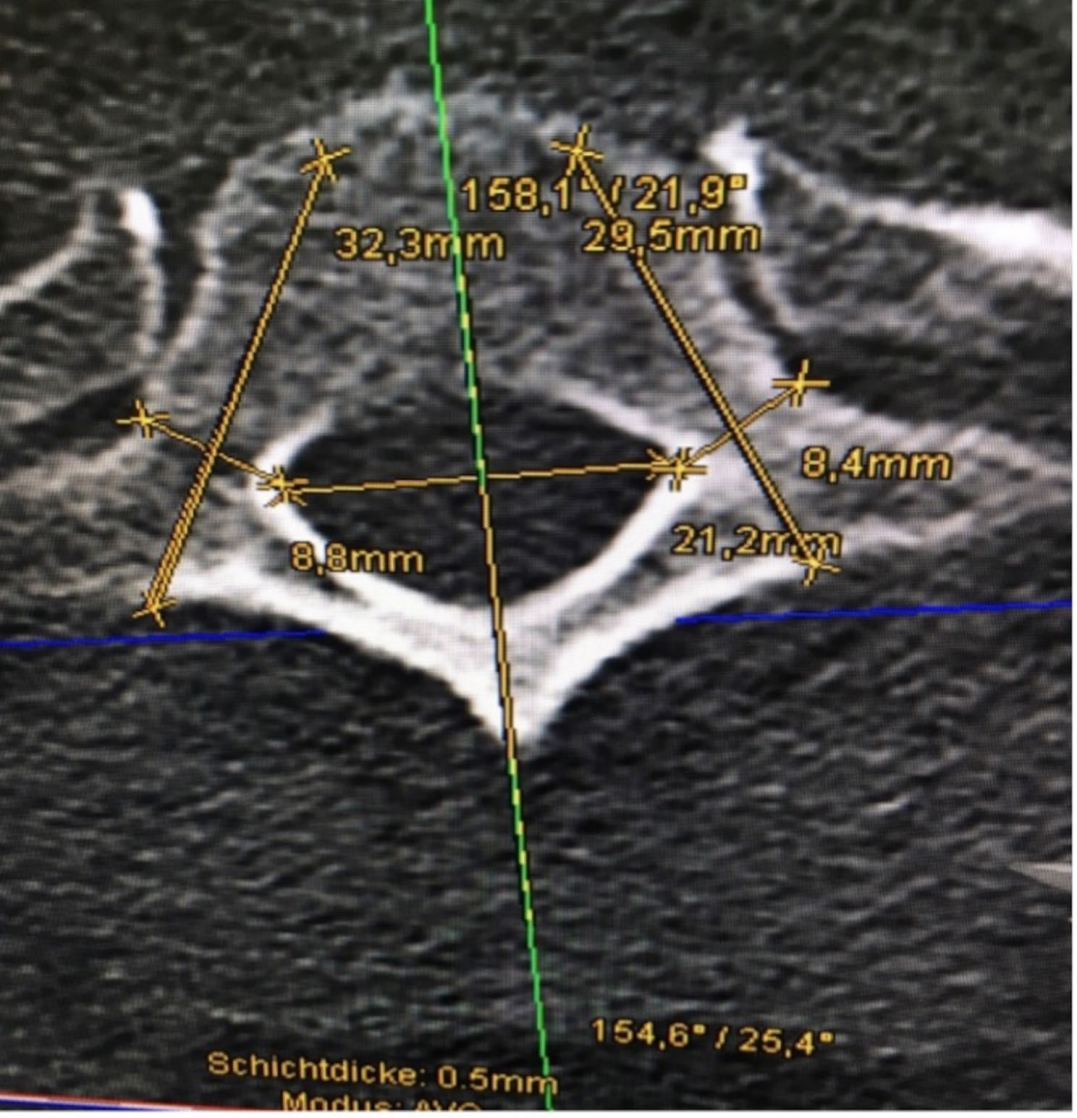
Thoracic vertebra in the axial plane with measurement of the pedicles and possible screw placement. The measurements of IPDI, OPW, PAL, PTA, PAA, and PAL are shown

**Fig. 3:**
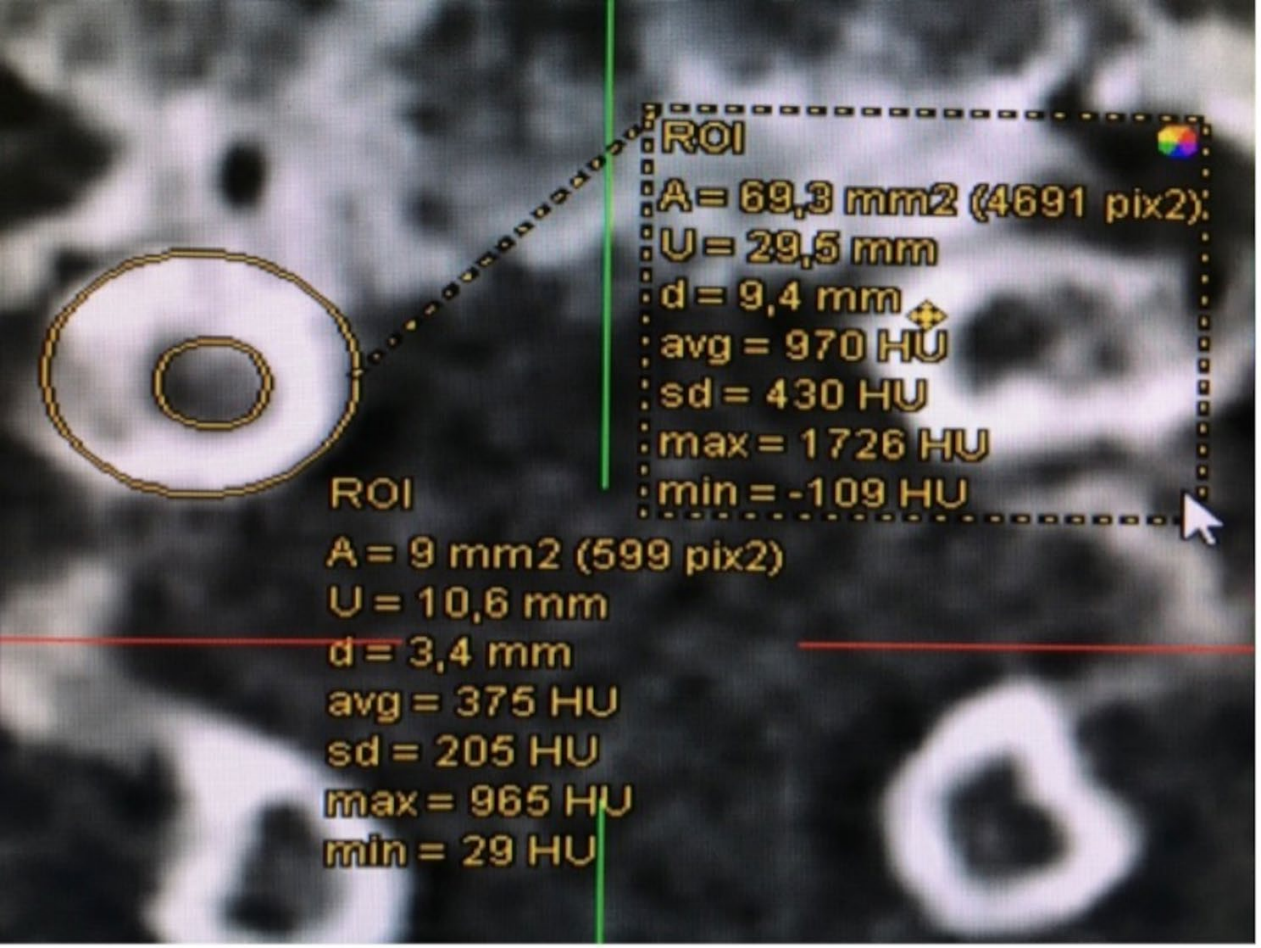
Inner and outer circumference of a pedicle in the coronary plain. Measurement of the IPD and OPD

**Fig. 4:**
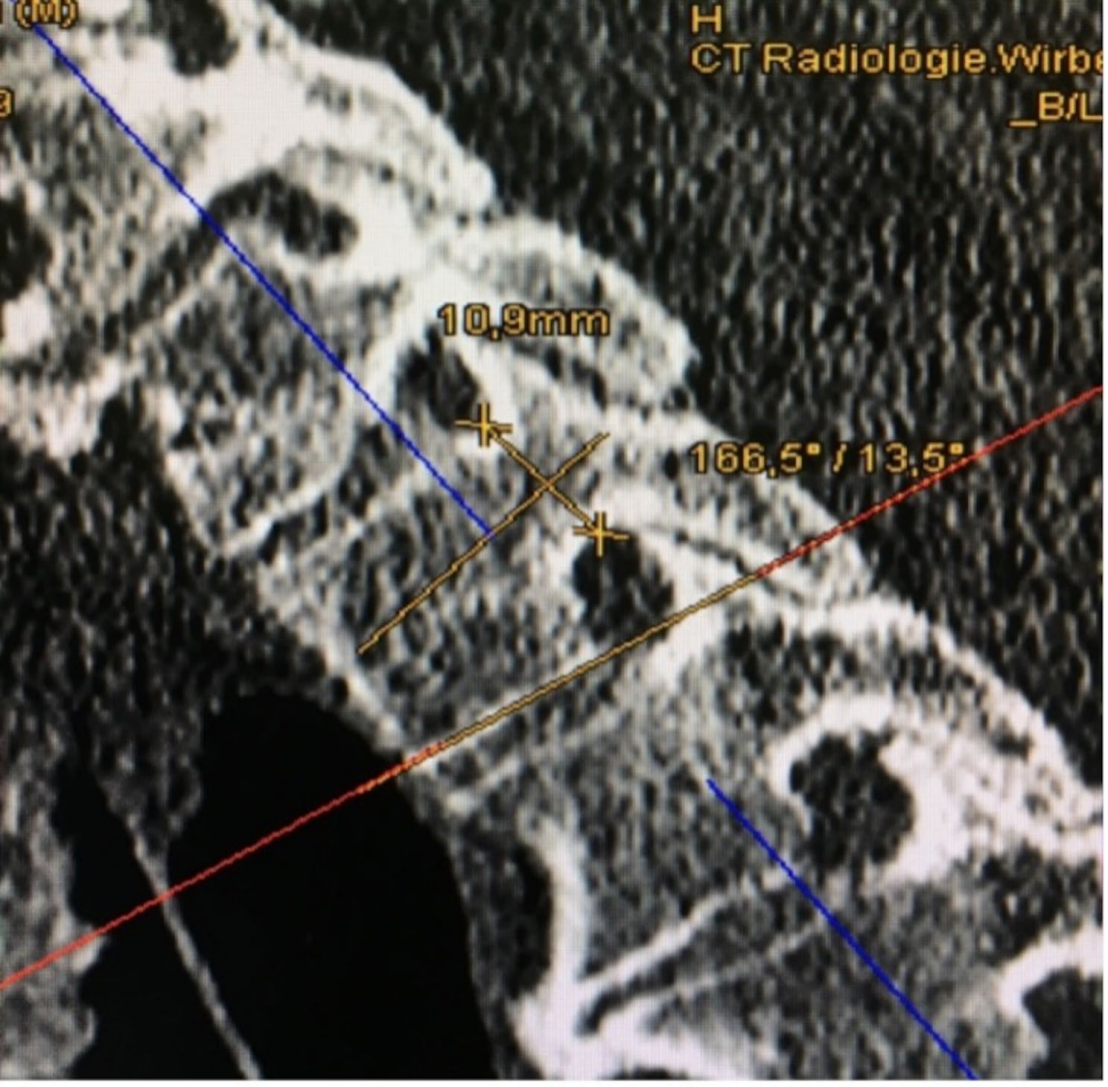
Pedicle measurements of the thoracic spine in the sagittal plane. The measurements of PSA and PH are shown

In this study, we retrospectively measured the thoracic spine of a total of 16 males and 16 females thoracic spines totaling in 768 individual pedicles. The patients were all of European ethnicity and were recruited retrospectively in the timeframe between August 2015 and August 2016. The CT slice thickness was a maximum of 1 mm in each scan used for the evaluation. None of the patients had health issues related to the measurement of the pedicles. This was guaranteed by looking at the indications for the CT images, reading the physician’s letters of the patients and finally by checking the CT images themselves. The exclusion criteria for the patients were a history of surgical intervention on the thoracic spine, a CT slice thickness of > 1 mm, pedicle fractures, vertebral body fractures, severe degenerative changes, neoplasms, severe osteoporosis, or osteoporotic fractures. Therefore, even if no records of previous surgeries were found and metallic objects were observed within any of the twelve thoracic vertebrae, the patient was immediately excluded from the study. This made finding enough patients a difficult task, since few patients undergo CT imaging of the entire thoracic spine without a past operation or health issues connected with the pedicles which would result in the patient being excluded. The patient pool was mostly filled with trauma patients who received imaging to exclude fractures of the spine. The scanner used at the institution during the time of the scans was an iCT Philips (Philips Medical Systems DMC GmbH, Hamburg, Germany) 256 slice detector (120 kV). This underlines the importance of morphometric understanding of the pedicles, since they are directly connected to the indication for CT imaging. Measurements were performed using Impax^™^ software, version 6.3 (AGFA HealthCare, Mortsel Belgium). We used the reconstruction options in all planes, which provided a good understanding of the structures of the pedicles. Furthermore, the software allowed specific configuration of the planes (beyond standard axial, sagittal, and coronal planes) to assess the pedicle morphology precisely. The pedicles were measured independently by two orthopaedic residents. In the event of deviating measured values, these were checked by a third measurement. The Figs. 1, 2, 3 and 4 show examples in measuring the abovementioned parameters.

### Measurements

The following parameters were measured on each pedicle of each vertebra following a standardized method:

The inner pedicle distance (IPDI), outer pedicle width (OPW), pedicle height (PH), pedicle axial length (PAL), inner and outer pedicle diameter (IPD/OPD), difference between the IPD and OPD (Diff), cortical thickness at different locations (CTcranial/CTmedial/lateral/CTcaudal/CTlateral/medial), maximal and minimal cortical thickness (CTmax/CTmin), pedicle sagittal and axial angle (PSA/PAA), and pedicle axial length (PAL) were measured.

Cortical thickness was measured from the axial plane’s perspective at the cranial, caudal, medial, and lateral portions of the pedicle and is illustrated in Fig. 1. CTmax and CTmin were either included in the first four measurements or measured separately and given a number representing the hand of a clock (for example 90° representing 3:00 o’clock). The axial plane, as shown in Fig. 2, allowed measurement of the IPD and OPD. The axial plane was realigned for each pedicle, ensuring measurement at the thickest point and ensuring that the pedicle was perpendicular to the plane. The green and red lines mark the sagittal and transverse planes, respectively. For the measurements of the IPDI, OPW, PAL and the PAA, the sagittal line was aligned with the processus spinosus of each vertebra. After that, the transverse plane was relocated to the height at which the pedicles reached their maximum diameter, at which point the measurements began. An example of this is shown in Fig. 3. The PAA was measured between the sagittal plane and the pedicles axis, after which PAL was measured considering the diameter of the screw that would be used on this vertebra. OPW was measured at an angle of approximately 90° to the PAL. Figure 4 shows the last measurement, taken from the perspective of the sagittal plane. Here you can identify a new, blue line representing the axial plane from which we were looking from earlier. The transverse plane was realigned each time with the base of the corpus vertebrae, starting from the median axis of the entire pedicle. The PSA was then measured in between the transverse plane and possible screw placement in the left and right pedicles. The PH can also be measured in this plane.

All measurements were registered allowing calculations and highlighting of the arithmetic medium, maximum, and minimum values as well as the standard deviation. After checking for a normal distribution, an unpaired t test was performed to check for significant changes. A significance level of *p* < 0.05 was chosen.

## Results

The age of the European patient cohort ranged from 41 to 92 years, with an average of 63.9 years ± 17.98 years (mean ± standard deviation, SD) when CT was performed.

### Outer and inner pedicle diameters

We analyzed the data to determine whether sex-specific differences could be observed.

The absolute minimum value was observed at thoracic vertebra four (T4), with an OPD of 5.6 mm (± 1.2 mm). The first maximum value for the OPD was located at thoracic vertebra T1 with value 8.7 mm (± 3 mm), and the second maximum was at T11 with an equal value of 8.7 mm (± 1.7 mm) on average. The maximum IPD occurs at T11 with a value of 5.1 mm (± 1.4 mm) and the minimum occurs at T4 with a value of 2.7 mm (± 0.9 mm).

With an average Diff of 3,3 mm (± 0,7 mm) for males and Diff of 3,4 mm (± 0,9 mm) for females, *p* = 0,19 statistically significant results could not be detected.

*Diagram 2* graphically demonstrates when the IPD is subtracted from the OPD, resulting in differences in the outer and inner diameters (Diff) of each pedicle. This value was calculated to approximate how much cortical bone is in each pedicle. In this diagram, we can observe an absolute maximum at T1 with a value of 4,3 mm (± 0,8 mm), an absolute minimum at T4 with a value of 2,9 mm (± 0,7 mm) and then a steady growth of the curve toward T11 where another local maximum is found with a value of 3,7 mm (± 0,8 mm). At T12, the difference between the outer and inner circumferences and thus the amount of cortical bone decreased again. Although this is only a curve that was derived and not directly measured, it is displayed and highlights T4 once again.

To gain some understanding of how the Diff (ri)/(le) corticalis thickness was located and distributed in each pedicle, *Diagram 2* shows the cortical thickness of the right and left pedicles across the thoracic spine.

### Lateral and medial walls

The average thickness of the lateral walls of the pedicle was 1.57 mm (± 0.52 mm). The absolute minimum cortical thickness was located at T4. The medial corticalis thickness is, on average, greater at 1,72 mm (± 0,42 mm). The smallest medial corticalis was located at T4, at 1.53 mm (± 0.33 mm). The smallest lateral corticalis was also located at T4, at 1,31 mm (± 0,31 mm). The lateral cortical bone was significantly thinner than the medial cortical bone (*p* < 0,001). An overview of the values for medial and lateral wall thickness can be seen in Table 1.

**Table 1:** Cortical thickness of medial and lateral walls

Vertebra	Medial in mm	± SD	Lateral in mm	± SD	*p* - value
T1	2.17	0.47	2.04	0.66	0.16
T2	1.88	0.40	1.60	0.40	< 0.001
T3	1.64	0.36	1.36	0.38	< 0.001
T4	1.53	0.33	1.31	0.31	0.001
T5	1.58	0.33	1.39	0.41	0.012
T6	1.57	0.36	1.51	0.44	0.260
T7	1.53	0.32	1.51	0.40	0.383
T8	1.59	0.35	1.51	0.37	0.191
T9	1.62	0.30	1.61	0.47	0.404
T10	1.72	0.38	1.76	0.62	0.363
T11	1.89	0.49	1.65	0.68	< 0.001
T12	1.92	0.43	1.55	0.56	< 0.001
Average	1.57	0.52	1.72	0.42	< 0.001

### Angle measurements of potential pedicle screws

The PSA, as shown in Table 2, for the male population was 17.4° (± 4.6° SD), and it was 14.9° (± 3.9° SD) for the female patients. Significant differences were observed (*p* < 0,001). In contrast, no significant sex-specific differences (*p* = 0,21) were detected for PAA. Here, the male average PAA was 14.1° (± 7.3°), and the female average was 13.5° (± 5.5°).

**Table 2:** Significant differences in PSA between male and female vertebrae

Vertebra	PSA				Vertebra	PSA			
	Right	± SD	Left	± SD		Right	± SD	Left	± SD
**T1**	*p* < 0.001				**T7**	*p* < 0.001			
Overall	12.13	3.90	12.66	3.39	Overall	19.24	4.87	19.05	4.39
Male	14.23	4.67	14.25	4.33	Male	21.98	6.19	21.01	5.28
Female	10.03	3.12	11.07	2.44	Female	16.49	3.55	17.08	3.49
**T2**	*p* = 0.006				**T8**	*p* = 0.009			
Overall	16.95	3.53	15.96	2.92	Overall	17.21	4.55	17.26	4.24
Male	18.39	3.85	16.81	2.96	Male	18.74	5.16	18.71	4.86
Female	15.50	3.20	15.11	2.88	Female	15.68	3.94	15.80	3.61
**T3**	*p* = 0.083				**T9**	*p* = 0.383			
Overall	16.64	4.51	15.84	3.88	Overall	15.86	3.66	16.11	3.92
Male	17.68	4.72	16.62	4.40	Male	15.93	3.61	16.86	4.31
Female	15.59	4.29	15.04	3.35	Female	15.78	3.71	15.35	3.53
**T4**	*p* = 0.083				**T10**	*p* = 0.147			
Overall	16.64	4.51	15.84	3.88	Overall	15.46	3.19	15.89	3.46
Male	17.68	4.72	16.63	4.40	Male	15.89	5.53	16.66	3.29
Female	15.59	4.29	15.04	3.35	Female	15.02	2.84	15.11	3.63
**T5**	*p* = 0.035				**T11**	*p* = 0.001			
Overall	16.93	4.35	16.99	3.96	Overall	14.17	3.36	14.61	3.57
Male	18.25	4.59	17.87	3.75	Male	15.71	3.33	16.10	3.68
Female	15.60	4.10	16.10	4.16	Female	12.63	3.38	13.11	3.46
**T6**	*p* = 0.001				**T12**	*p* = 0.073			
Overall	18.22	3.91	18.49	3.48	Overall	13.04	4.14	13.42	4.43
Male	19.91	4.77	20.17	4.62	Male	15.01	3.43	15.76	4.18
Female	16.53	3.05	16.81	2.34	Female	11.07	4.85	11.07	4.68

Finally, measurements of the PAL, representing the lengths of the screws used in surgery, showed significant sex-specific differences. The male population had an average PAL of 43.6 mm (± 6.4 mm), whereas the females had an average PAL of 39.6 mm (+- 6.5 mm) (*p* < 0.001). The PAL increased throughout the thoracic spine, with the smallest increase occurring at T1 (31.8 mm (± 3.5 mm)) and the greatest increase occurring at T12 (47.4 mm (± 4.8 mm)). The PAL is shown in Table 3.

**Table 3:** Significant differences in PAL between male and female vertebrae

Vertebra	PAL				Vertebra	PAL			
	Right	± SD	Left	± SD		Right	± SD	Left	± SD
**T1**	*p* < 0.001				**T7**	*p* < 0.001			
Overall	32.06	3.28	31.52	3.65	Overall	44.46	4.42	43.96	4.56
Male	33.55	3.21	32.93	3.06	Male	47.01	2.94	46.45	2.69
Female	30.57	2.67	30.10	3.73	Female	41.91	4.21	41.48	4.75
**T2**	*p* < 0.001				**T8**	*p* < 0.001			
Overall	33.67	3.04	33.69	3.61	Overall	45.63	4.64	44.48	4.63
Male	34.69	2.71	35.58	3.02	Male	48.10	3.85	47.21	3.18
Female	32.64	3.09	31.81	3.19	Female	43.15	4.08	41.91	4.35
**T3**	*p* < 0.001				**T9**	*p* < 0.001			
Overall	35.69	3.46	36.31	4.23	Overall	45.90	4.19	46.05	4.89
Male	37.34	3.33	38.61	3.64	Male	48.21	3.43	48.61	3.57
Female	34.04	2.80	34.02	3.55	Female	43.60	3.65	43.50	4.77
**T4**	*p* < 0.001				**T10**	*p* = 0.002			
Overall	38.27	4.59	38.27	4.74	Overall	46.74	4.71	46.30	4.99
Male	40.44	4.21	40.96	3.34	Male	48.46	4.64	48.23	4.59
Female	36.11	3.96	35.58	4.45	Female	45.01	4.24	44.38	4.75
**T5**	*p* < 0.001				**T11**	*p* < 0.001			
Overall	40.69	4.24	40.82	4.48	Overall	47.33	4.23	47.09	3.87
Male	43.31	2.92	43.49	3.27	Male	49.07	4.09	49.06	3.01
Female	38.08	3.76	38.14	3.93	Female	45.60	3.73	45.12	3.70
**T6**	*p* < 0.001				**T12**	*p* = 0.215			
Overall	42.85	4.15	41.92	4.42	Overall	47.08	5.07	47.68	4.65
Male	45.10	2.47	44.36	2.08	Male	47.45	6.08	48.82	4.41
Female	40.45	4.28	39.31	4.82	Female	46.71	4.00	46.54	4.75

The average outer pedicle width (OPW) shown in Table 4 was 6.87 mm (± 1.65 mm) for males and 5.89 mm (± 1.52 mm) for females, with a significant difference between the two (*p* < 0.001).

**Table 4:** Significant differences in the outer pedicle width (OPW) between male and female thoracic vertebrae

Vertebra	OPW				Vertebra	OPW			
	Right	± SD	Left	± SD		Right	± SD	Left	± SD
**T1**	*p* = 0.008				**T7**	*p* = 0.002			
Overall	8.26	1.29	8.52	1.38	Overall	5.58	1.14	5.52	1.01
Male	8.71	1.22	9.02	1.45	Male	6.01	1.17	5.93	0.98
Female	7.81	1.23	8.02	1.14	Female	5.16	0.95	5.11	0.88
**T2**	*p* < 0.001				**T8**	*p* = 0.001			
Overall	6.99	1.12	6.93	1.20	Overall	5.91	1.00	5.68	1.10
Male	7.60	0.99	7.52	1.01	Male	6.32	0.95	6.18	0.83
Female	6.38	0.91	6.34	1.10	Female	5.50	0.91	5.22	1.14
**T3**	*p* = 0.027				**T9**	*p* = 0.002			
Overall	5.61	0.73	5.73	1.14	Overall	6.23	1.21	6.18	1.14
Male	5.90	0.49	6.01	1.14	Male	6.74	1.17	6.61	0.87
Female	5.32	0.83	5.44	1.09	Female	5.71	1.04	5.74	1.24
**T4**	*p* = 0.038				**T10**	*p* = 0.003			
Overall	5.15	0.95	5.31	0.96	Overall	6.93	1.32	6.90	1.53
Male	5.39	0.78	5.62	0.78	Male	7.47	1.23	7.44	1.54
Female	4.91	1.06	5.00	1.05	Female	6.39	1.21	6.36	1.35
**T5**	*p* = 0.032				**T11**	*p* = 0.006			
Overall	5.21	1.24	5.15	0.98	Overall	7.51	1.88	7.51	1.76
Male	5.53	1.23	5.46	0.87	Male	8.34	1.67	7.99	1.59
Female	4.89	1.20	4.84	1.00	Female	6.67	1.73	7.03	1.84
**T6**	*p* = 0.001				**T12**	*p* < 0.001			
Overall	5.33	1.11	5.48	0.90	Overall	7.87	2.11	7.64	2.03
Male	5.74	1.19	5.86	0.91	Male	9.11	1.48	8.38	1.65
Female	4.89	0.83	5.09	0.72	Female	6.62	1.93	6.89	2.15

Similarly, the pedicle height (PH) shown in Table 5 was significantly greater for male patients (12.86 ± 2.46 mm) than for female patients (11.77 ± 2.46 mm), with a *p* value < 0.001.

**Table 5:** Significant differences in pedicle height (PH) between male and female thoracic vertebrae

Vertebra	OPW				Vertebra	OPW			
	Right	± SD	Left	± SD		Right	± SD	Left	± SD
**T1**	*p* = 0.004				**T7**	*p* = 0.016			
Overall	9.07	1.31	9.22	1.25	Overall	11.71	0,84	11,65	1,33
Male	9.46	1.52	9.73	1.45	Male	12.09	0,64	11,93	1,30
Female	8.67	0.96	8.71	0.77	Female	11.33	0,85	11,38	1,34
**T2**	*p* = 0.001				**T8**	*p* < 0.001			
Overall	10.32	1.29	10.26	1.25	Overall	12.04	1,40	11,84	1,23
Male	10.82	1.40	10.78	1.16	Male	12.84	1,40	12,48	1,28
Female	9.82	0.96	9.74	1.15	Female	11.24	0,87	11,24	0,85
**T3**	*p* < 0.001				**T9**	*p* = 0.002			
Overall	11.23	1.36	11.20	1.28	Overall	12.87	1,59	12,93	1,49
Male	11.94	1.38	11.74	1.12	Male	13.46	1,46	13,46	1,38
Female	10.51	0.91	10.67	1.23	Female	12.27	1,53	12,39	1,44
**T4**	*p* = 0.002				**T10**	*p* = 0.001			
Overall	11.08	1.32	11.13	1.40	Overall	14.58	1,40	14,60	1,62
Male	11.58	1.37	11.68	1.27	Male	15.02	1,32	15,38	1,46
Female	10.59	1.08	10.59	1.34	Female	14.13	1,37	13,81	1,41
**T5**	*p* < 0.001				**T11**	*p* = 0.037			
Overall	11.23	1.40	11.03	1.31	Overall	16.02	1,52	15,91	1,52
Male	11.89	1.38	11.93	0.77	Male	16.23	1,81	16,48	1,21
Female	10.58	1.10	10.14	1.12	Female	15.81	1,20	15,33	1,62
**T6**	*p* < 0.001				**T12**	*p* = 0.017			
Overall	11.52	1.28	11.55	1.28	Overall	16.17	1,45	16,42	1,33
Male	12.17	1.08	12.14	0.99	Male	16.67	1,38	16,74	1,47
Female	10.83	1.12	10.93	1.28	Female	15.68	1,37	16,09	1,13

A summarized overview of the sex-specific differences in the thoracic spine is provided in Table 6.

**Table 6:** Summarized sex-specific differences in vertebral anatomy

Measurement	Male	± SD	Female	± SD	*p* - value
Diff	3.3 mm	0.7 mm	3.4 mm	0.9 mm	0.19
PH	12.86 mm	2.46 mm	11.77 mm	2.46 mm	< 0.001
OPW	6.87 mm	1.65 mm	5.89 mm	1.52 mm	< 0.001
PAL	43.6	6.4 mm	39.6 mm	6.5 mm	< 0.001
PSA	17.4 °	4.6 °	14.9 °	3.9 °	< 0.001
PAA	14.1 °	7.3 °	13.5 °	5.5 °	0.21

## Discussion

The results of the measurements show that the OPD and IPD change in a similar way throughout the thoracic spine, leaving the cortical bone with a constant thickness. Variability in the OPD and IPD therefore originates from an increase in the total amount of Substantia spongiosa, decreasing the stability of the pedicles because relatively more of the pedicle is made up of spongiosa rather than the more stable corticalis. As a tradeoff, here, larger pedicle screws could be used, and correct placement is not as difficult to achieve. On the other hand, more stable, smaller pedicles are prone to more screw misplacement and are therefore also at risk. The rate of screw misplacement for pedicles with an OPD smaller than 5 mm is 33% [18].

For screw placement, the greatest interest of this study was minimal corticalis thickness since pedicle screws are more likely to damage surrounding areas by perforating the pedicle when the corticalis is thinner.

Moreover, the thinnest cortical wall portions of the pedicles were measured laterally, with an average of 1.57 mm (± 0.52 mm). This allows the following conclusion: when operating on a pedicle of the thoracic spine, the greatest risk for a screw to penetrate the wall of the pedicle and damage surrounding structures is in the lateral wall. The increasing spongiosa thickness in some parts of the thoracic spine pedicles does not protect against such perforation because the spongiosa is built to withstand pressure from on top and below and not the lateral forces a screw would impose when perforating the pedicle [19]. Structures that lie laterally from the pedicles of the thoracic spine include the spinal nerve, facet joints and a large blood vessel plexus [20].

Furthermore, the medial cortical wall was significantly thicker than the lateral portions by 1.72 mm (± 0.42 mm) on average. Although the corticalis on the medial portions was slightly thicker than that on the lateral portions, structures near the medial portion, such as the spinal canal in which the spinal cord is found of the pedicle, are more fragile and could have a more disastrous effect on the patient when damaged. Therefore, the risk of medial perforation by a pedicle screw should not be underestimated.

Interestingly, in this study, both the medial and lateral walls of the pedicle were most vulnerable at T4, which could indicate that screw placement in this segment is especially demanding.

### Limitations

This study has several Limitations. The average age of the patients was 63.9 years, with a wide range between 41 and 92 years. Since thoracic spine morphology may differ with age, particularly due to degenerative changes, this age range may not accurately represent the anatomy of younger patients undergoing pedicle screw fixation. The exclusion of individuals with severe osteoporosis or other health issues limits the study’s applicability to elderly patients with these common conditions. Furthermore, being a retrospective study, it is limited by the availability of existing CT scans and associated data, which may introduce biases related to patient selection and data collection methods. Addressing these limitations in future studies could improve the generalizability, applicability, and clinical relevance of the findings.

## Conclusion

Finally, correct screw placement in pedicles is essential achieving long-term benefits after surgery. This study aimed to improve the understanding of pedicle placement in the thoracic spine and recommended the use of 3D-reconstruction programs for each surgical measure that involves screw placement. Especially in segment T4, the screw length, diameter, and sagittal and axial angles must be corrected to reduce the risk for complications.

## Data Availability

No datasets were generated or analysed during the current study.
